# Unwarranted variation of coronary stent choice in The Netherlands

**DOI:** 10.1007/s12471-015-0781-7

**Published:** 2016-01-13

**Authors:** S.C.A.M. Bekkers, J.C.A. Hoorntje

**Affiliations:** Department of Cardiology, Maastricht University Medical Center, P. Debyelaan 25, 6202 AZ PO Box 5800, Maastricht, The Netherlands

The primary aim of treating patients with coronary artery disease (CAD) is to reduce symptoms and improve prognosis, which encompasses risk factor modification and pharmacological management. Coronary artery bypass grafting (CABG) and percutaneous coronary intervention (PCI) using bare-metal stents (BMS) and drug-eluting stents (DES) have become established additional treatment options. PCI is undoubtedly the preferred reperfusion therapy for ST-elevation myocardial infarction (STEMI), but optimal treatment for complex non-STEMI and stable CAD is less well established. Whether optimal medical therapy, PCI, or CABG is preferred in this complex group of patients depends on the risk-benefit ratios of these treatment strategies and should be discussed in multidisciplinary teams (Heart Team) [1]. While DES are more effective in reducing restenosis than BMS and target-vessel revascularisation (TVR, estimated reduction 50–70 %), DES are more costly and require prolonged dual antiplatelet therapy that increases bleeding complications. The benefits of DES use are greatest in patients at the highest risk of TVR [2].

Ideally, a country’s healthcare system should guarantee that all patients receive optimal medical care, independent of their welfare status or whatever hospital they are referred to. When standards of care are clear, there should be no variation in practice across the country. With the expansion of interventional centres in the Netherlands a rather complete geographical spread has been reached, but whether treatment disparities exist between centres has largely been unknown. In the current issue of the Netherlands Heart Journal, a study by Burgers et al. [3] investigated the treatment variation in stent choice (BMS vs. DES) in patients with stable and unstable CAD across 4 medical centres in the Netherlands. This study is a sub-analysis of the CIRCULATING CELLS study that included patients from March 2009 until September 2011 and aimed to discover markers to predict future cardiovascular events. Overall, 66 % of 442 patients were treated with DES with no difference of DES use between stable (*n* = 358) or unstable CAD patients (*n* = 84). Increased use of DES was associated with NYHA class, smoking, multivessel disease, previous PCI, diabetes mellitus, and interestingly treating hospital which explained 33 % of the variation in stent choice. DES use in patients varied widely between hospitals, ranging from 50 to 99 %. The increased use of DES in certain patient groups largely followed the guidelines, because DES is considered most useful in patients at risk for restenosis. However, the variation of DES use between hospitals is curious and not immediately explained by differences in patient case mix. What then, explains this unwarranted variation in stent choice?

To understand unwarranted variation in medical practice, different areas need to be considered [4]. First, underuse of effective (evidence-based) PCI care will likely be small but cannot be ignored despite appropriate training and skills. It is known that underuse of evidence-based medicine can be attenuated when doctors practice team medicine, i.e. in a Heart Team. Providing and adapting state-of-the-art care in large rural areas is more difficult than in densely populated urban areas. Second, the largest variation may be explained by preference-sensitive care. Here, more than one generally accepted treatment option is available and the variation largely depends on differences in opinion between cardiologists. Because DES are so effective in reducing restenosis, many cardiologists have adopted DES for ‘off-label’ indications and ‘consume’ more DES than others. Here, patient preferences and shared decision-making have shown to improve the quality of care and attenuate medical practice variation. Third, supply-sensitive care describes the available resources that may differ between hospitals as a result of differing hospital contracts with health insurance companies, stent producing companies, budgetary constraints, financial incentives, etc.

Recruitment of study patients took place during a transition period of advanced and more expensive DES technology with newer drug coatings that gradually and increasingly replaced BMS. Because the study spans only 2 years, differences between cardiologists as either *early adopters* or *late believers* of new technology more clearly affect differences in stent choice than when a longer period was studied. As it happens over time and when evidence and experience have sufficiently been build up, differences between cardiologists, hospitals and material costs are likely to decrease.

Nonetheless, Burgers et al. are to be congratulated, because they are the first to show a hospital-dependent variation of DES use in the Netherlands, where geographical differences should not be an issue. Hospital differences in DES use have been shown in other parts of the world as well. In the United States, DES use has been shown to vary widely among physicians with only a modest correlation to patients’ risk of restenosis [5]. Reducing DES use among patients at low TVR risk may substantially lower healthcare costs with only a minimal increase in TVR. However, before jumping to conclusions, an important question remains unanswered: does practice variation matter? Knowing whether hospital and geographical variations in the use of DES affects outcome would help in defining the most optimal treatment strategy that improves prognosis and is cost-effective. With this study, an important first step has been taken by creating awareness of regional variations in stent choice in the Netherlands. We now have to take this message one step further and investigate whether this affects outcome.

## Funding

None.

## Conflicts of interest

None declared.
